# Common Variants Coregulate Expression of *GBA* and Modifier Genes to Delay Parkinson's Disease Onset

**DOI:** 10.1002/mds.28144

**Published:** 2020-06-18

**Authors:** William Schierding, Sophie Farrow, Tayaza Fadason, Oscar E.E. Graham, Toni L. Pitcher, Sara Qubisi, Alan J. Davidson, Jo K. Perry, Tim J. Anderson, Martin A. Kennedy, Antony Cooper, Justin M. O'Sullivan

**Affiliations:** ^1^ Liggins Institute The University of Auckland Auckland New Zealand; ^2^ Gene Structure and Function Laboratory, Department of Pathology and Biomedical Science University of Otago Christchurch New Zealand; ^3^ Department of Medicine University of Otago Christchurch New Zealand; ^4^ Brain Research New Zealand The University of Auckland Auckland New Zealand; ^5^ New Zealand Brain Research Institute Christchurch New Zealand; ^6^ Department of Molecular Medicine and Pathology, School of Medical Sciences The University of Auckland Auckland New Zealand; ^7^ Neurology Department Christchurch Hospital, Canterbury District Health Board Christchurch New Zealand; ^8^ Australian Parkinsons Mission Garvan Institute of Medical Research Sydney New South Wales Australia; ^9^ St Vincent's Clinical School University of New South Wales Sydney New South Wales Australia

## Abstract

**Background:**

*GBA* mutations are numerically the most significant genetic risk factor for Parkinson's disease (PD), yet these mutations have low penetrance, suggesting additional mechanisms.

**Objectives:**

The objective of this study was to determine if the penetrance of *GBA* in PD can be explained by regulatory effects on *GBA* and modifier genes.

**Methods:**

Genetic variants associated with the regulation of *GBA* were identified by screening 128 common single nucleotide polymorphisms (SNPs) in the *GBA* locus for spatial *cis‐*expression quantitative trail locus (supported by chromatin interactions).

**Results:**

We identified common noncoding SNPs within *GBA* that (1) regulate GBA expression in peripheral tissues, some of which display α‐synuclein pathology and (2) coregulate potential modifier genes in the central nervous system and/or peripheral tissues. Haplotypes based on 3 of these SNPs delay disease onset by 5 years. In addition, SNPs on 6 separate chromosomes coregulate *GBA* expression specifically in either the substantia nigra or cortex, and their combined effect potentially modulates motor and cognitive symptoms, respectively.

**Conclusions:**

This work provides a new perspective on the haplotype‐specific effects of *GBA* and the genetic etiology of PD, expanding the role of *GBA* from the gene encoding the β‐glucocerebrosidase (GCase) to that of a central regulator and modifier of PD onset, with *GBA* expression itself subject to distant regulation. Some idiopathic patients might possess insufficient *GBA*‐encoded GCase activity in the substantia nigra as the result of distant regulatory variants and therefore might benefit from *GBA*‐targeting therapeutics. The SNPs’ regulatory impacts provide a plausible explanation for the variable phenotypes also observed in *GBA*‐centric Gaucher's disease and dementia with Lewy bodies. © 2020 The Authors. *Movement Disorders* published by Wiley Periodicals, LLC on behalf of International Parkinson and Movement Disorder Society.

Parkinson's disease (PD) is a progressive neurodegenerative condition pathologically characterized by the presence of α‐synuclein (αsyn) containing Lewy bodies.^1^, ^2^, ^3^ The defining motor symptoms of PD are associated with the loss of dopaminergic neurons in the substantia nigra, but patients can also suffer numerous nonmotor symptoms that include cognitive impairment. The disease affects more than 7 million individuals worldwide,^4^, ^5^ and the rising incidence of PD will increase the burden of this incurable disease on healthcare systems.^6^ The current therapy for PD provides only symptomatic relief with no therapeutics demonstrated to attenuate disease progression, although the potential for drug discovery and/or repurposing is increasing as research continues to elucidate the pathways underlying PD pathogenesis.^7^, ^8^, ^9^, ^10^ PD is predominantly considered an idiopathic disease, but recent studies support a much stronger influence of genetic determinants in the idiopathic cases of PD (10%–30% heritability).^11^, ^12^ Among the ~90 genetic loci associated with PD, the *GBA* locus is the most common genetic risk factor with mutations in 2.3% to 9.4% (11%–31% in Ashkenazi Jews) of PD cases,^13^ with ethnicity playing a large role in determining the risk range.^14^ However, the estimated lifetime risk ratio of a *GBA* mutant carrier developing PD is only 21% to 29%, consistent with the variable penetrance of the *GBA* coding variants.^15^, ^16^ Furthermore, recent observations identified reduced *GBA*‐encoded protein, β‐glucocerebrosidase (GCase) activity in patients with idiopathic PD (IPD) in the absence of loss‐of‐function (LoF) *GBA* mutations.^17^, ^18^, ^19^ Collectively, these observations are consistent with regulatory impacts on *GBA* expression and other modifier genes contributing to PD development.

Cognitive decline is among the most frequent and debilitating nonmotor symptoms of patients with PD with 46% and 83% of surviving patients, at 10 and 20 years postdiagnosis, respectively, having progressed to dementia (PD dementia [PDD]).^20^, ^21^ Notably, patients who carry *GBA* mutations display higher frequencies of cognitive decline and 5 times the risk of progression to dementia relative to patients who do not have *GBA* mutations.^22^, ^23^, ^24^, ^25^ Postmortem studies indicate that cortical Lewy body pathology is a main pathological correlate of dementia in PD.^26^

Heterozygote *GBA* coding mutations are also a significant risk factor for dementia with Lewy bodies (DLB; odds ratio > 8), a disease that correlates closely with PDD but in which cognitive symptoms precede or coincide with motor deficits.^27^ As with PD, *GBA* mutations display incomplete penetrance in DLB for unknown reasons.^28^

*GBA* mutations are also the basis of Gaucher disease, an autosomal recessive disorder that can present with nonneurological or neuropathic forms. Genotype–phenotype studies reveal limited correlation; patients possessing the same *GBA* mutation present with different phenotypes, whereas clinically similar patients possess a diverse range of mutations.^29^ This phenotypic variation among Gaucher patients sharing the same *GBA* genotype further implicates the role of modifiers that could include *GBA* variants modulating *GBA* expression and/or potential modifier genes.^29^

Here we undertook an in‐depth characterization of the regulatory control of *GBA* and the identification of potential modifier genes. To identify the regulatory network responsible, we interrogated the *GBA* locus for regulatory interactions modulating expression of *GBA* and/or potential modifier genes. Our findings identified *GBA* expression as being regulated by *cis* elements (tagged by 56 single nucleotide polymorphisms [SNPs]) in tissues excluding the brain. Notably, 73 SNPs located within *GBA* affected the expression levels of 143 potential modifier genes. In contrast, GBA expression in the substantia nigra and cortex was regulated in *trans* from other chromosomes. Cohort analyses confirmed that individuals with minor allele variants at noncoding regulatory positions within *GBA* have a significant correlation with PD symptom onset and diagnosis. These observations reveal a genome‐wide regulatory network for *GBA,* providing insights into the mechanisms underlying the variable phenotypes and incomplete penetrance observed in patients with PD.

## Methods

### Variant Selection

We selected all common SNPs within the *GBA* locus chr1:155204239‐155214653 (dbSNP build151, appear in at least 1% of the global population). We added 1 rare SNP (rs2230288) in the coding region of *GBA* (chr1:155204239‐155214653) because it had a known *cis‐*expression quantitative trail locus (eQTL) with *GBA* in the Genotype‐Tissue Expression (GTEx) project (Supplementary Fig. 1). Genomic positions of SNPs are reported for human reference hg19.

### Identification of SNP‐Gene Spatial Relationships in the *GBA* Locus

For all SNPs in the *GBA* locus, putative spatial regulatory connections were identified. We used the contextualize developmental SNPs using 3D information (CoDeS3D) algorithm^30^ (https://github.com/Genome3d/codes3d-v1; Supplementary Fig. 1) to integrate both spatial interaction (Hi‐C data) plus expression data (eQTL) to identify genes whose transcript levels depend on the identity of the SNP (ie, spatial eQTL).^30^, ^31^

Hi‐C captures the spatial organization of chromatin by identifying regions of the genome that are physically associated and can therefore be covalently connected by a cross‐linking agent.^32^ Such spatial connections were identified from previously generated Hi‐C libraries of various origins (Supplementary Table 1): (1) cell lines GM12878, HMEC, HUVEC, IMR90, K562, KBM7, HELA, NHEK, and hESC (gene expression omnibus [GEO] accession numbers GSE63525,^33^ GSE43070,^34^ and GSE35156^35^); (2) tissue‐specific data from the encyclopedia of DNA elements (ENCODE) sourced from the adrenal gland, bladder, dorsolateral prefrontal cortex, hippocampus, lung, ovary, pancreas, psoas muscle, right ventricle, small bowel, and spleen (GSE87112)^36^; and (3) tissues of neural origin from the cortical and germinal plate neurons (GSE77565),^37^ cerebellar astrocytes, brain vascular pericytes, brain microvascular endothelial cells, SK‐N‐MC human neuroblastoma cell line, spinal cord astrocytes (GSE105194, GSE105513, GSE105544, GSE105914, GSE105957),^38^ and neuronal progenitor cells (GSE52457).^39^

To identify SNP locations in the Hi‐C data, all possible Hi‐C fragment locations were identified through digital digestion of the hg19 reference genome (matching the restriction enzyme from the Hi‐C libraries MboI or HindIII). First, we mapped SNPs to genome fragments (ie*,* SNP fragment). Next, all SNP fragments were queried against the Hi‐C databases to identify distal fragments of DNA that spatially connect to the SNP fragment. If the distal fragment contained the coding region of a gene, a SNP–gene spatial connection was confirmed. There was no binning or padding around restriction fragments to obtain gene overlap.

### Confirmation of SNP–Gene Regulatory Relationships in the *GBA* Locus

Spatial SNP–gene pairs were queried in the genotype‐tissue expression (GTEx) database (www.gtexportal.org, version 7^40^) to identify spatial SNP–gene pairs with significant eQTLs (both *cis‐*acting and *trans‐*acting eQTL; false discovery rate (FDR) adjusted *P* < 0.05; Supplementary Fig. 1). Because of the tissue specificity of the GTEx database, it is possible to assign tissue specificity to the transcription changes.

### Genome‐Wide Search for Distant Regulators of *GBA* Transcription

We performed a genome‐wide search of all 31,471,836 SNPs in dbSNP151 (as available in GTEx version 7^40^) for an association with *GBA* transcription (Supplementary Fig. 1). All SNPs suggestive of genome‐wide significance (*P* < 1 × 10 − ^6^) were also tested with the CoDeS3D^30^ algorithm to discover which other genes might be coregulated by the same SNPs.

### Gene Ontology, Pathway Analysis, and Functional Prediction

To identify overlapping biochemical functions and pathways, we queried the KEGG Pathway Database (https://www.kegg.jp/kegg/pathway.html). We used all genes identified as associating with the *GBA* locus, forming significant first‐level and second‐level pathway maps. We identified drugs that target the genes and drug mechanisms (Drug Gene Interaction database).

All identified genes were searched for known PD associations in the literature by Boolean searches of the literature databases (ie, Medline, EMBASE, Scopus, and Web of Science) using “[gene name] and Parkinson's,” “[gene name] and *GBA*,” or “[gene name] and alpha‐synuclein” as search terms.

### Haplotype Analyses

Variants in the *GBA* locus from 229 patients with PD (Table 1) were identified by Nanopore sequencing and confirmed via Sanger sequencing as previously described.^41^ In summary, FASTQ reads with a quality score (qscore) <7 were removed from the analysis. Porechop version 0.2.4 (https://github.com/rrwick/Porechop) was used to remove sequencing adapters and to demultiplex the reads. The reads were aligned against the genome reference consortium human build 38 (GRCh38) human reference genome using Minimap2 version 2.16 (https://lh3.github.io/minimap2/). Variant calling was performed using Nanopolish version 0.11.1 (default parameters; https://github.com/jts/nanopolish). Haplotypes were phased with WhatsHap version 0.18^42^ (default parameters), and haplotypes were determined using the haplotypes version 1.1 R package (R Foundation for Statistical Computing, Vienna, Austria). The 55 haplotypes identified and other clinical outcomes were used for further analysis. Haplotypes were then sorted into clusters A and B by genotypes (Euclidean distance, Ward clustering). Individuals were then assigned to a haplotype group based on the identity of their 2 haplotypes (homozygous haplotype cluster A, heterozygous, or homozygous haplotype cluster B). We regressed haplotype groups against phenotypic measures of IPD, assuming that haplotype relates to phenotype as an additive model (haplotype dosage effect). The additive model defines the effect of haplotype allele count, where the effect on phenotype is a linear combination of the haplotypes (the phenotype of heterozygous haplotype alleles is between the homozygous effect sizes).

**TABLE 1.** Demographic and clinical data for the Parkinson's disease patients in the New Zealand Brain Research Institute (NZBRI) cohort separated by *GBA* mutation status

Cohort Characteristic	Known GBA Mutation	No Known GBA Mutation	*P* Value
n	21	208	
Sex (% male)	71	67.3	0.81
Education, y	13.3 (3.1)	12.8 (2.6)	0.41
Follow‐up duration, y	5.8 (3.6)	5.1 (3.6)	0.397
Symptom onset age, y	58.3 (9.2)	60.7 (8.5)	0.222
Diagnosis age, y	60.1 (9.1)	62.6 (8.5)	0.203
Disease duration, y	15.2 (6.9)	13.7 (5.8)	0.268
Age at death, y	76.3 (8.4)	78.3 (6.1)	0.169
Age at dementia, y	71.3 (5.7)	74.8 (5.8)	0.117
Disease duration until dementia, y	10.8 (4.6)	10.0 (4.1)	0.627
Dementia, %	42.9 (95% CI, 24.6–63.5)	20.2 (95% CI, 15.3–26.2)	0.026

Values are mean (standard deviation) unless otherwise stated. Patients met the UK Brain Bank criteria for Parkinson's disease and were excluded as outlined in Graham et al.^41^ Reprinted with permission from *Parkinsonism & Related Disorders* 2020;70:36–41, © 2019 Elsevier Ltd.

Abbreviation: CI, confidence interval.

## Results

### Genetic Variants at the *GBA* Locus Regulate *GBA* Expression in Peripheral Tissues

**FIG. 1.**
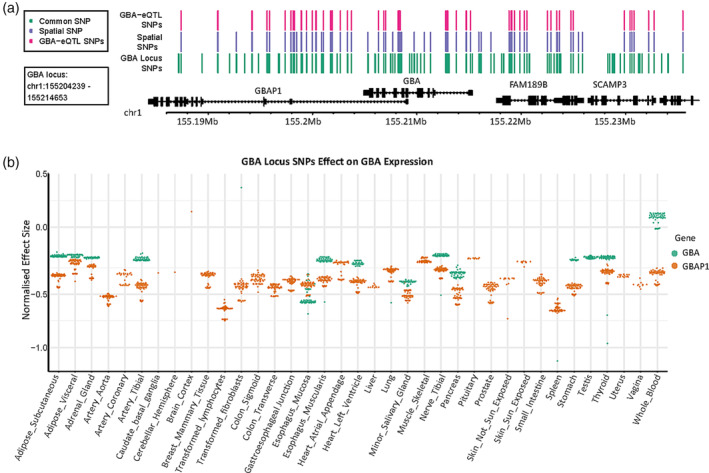
*GBA* is subject to tissue‐specific *cis* acting spatial regulation. (**A**) The *GBA* locus (*GBA* GENCODE coding region ± 20 kb on either side) contains 128 common SNPs (green lines); 57% (73) have significant SNP–gene associations to either/both local genes in 1q21 as well as genes across many other chromosome (purple lines; Supplementary Table 2, Supplementary Figure 2a), and 44% (56) have significant eQTLs with *GBA* (pink lines). (**B**) SNPs within the *GBA* locus have a varied effect on the expression of *GBA* (green) and the pseudogene *GBAP1* (orange). Previous findings in *GBA* have noted a strong downregulation of *GBA* in disease pathways, which is consistent with the findings here that most *GBA* locus variants downregulate both *GBA* and *GBAP1*. However, blood is an exception. eQTL, expression quantitative trail locus; SNPs, single nucleotide polymorphisms. [Color figure can be viewed at wileyonlinelibrary.com]

The substantial incomplete penetrance of *GBA* mutations motivated us to identify genetic variants at the *GBA* locus that regulate *GBA* expression in human tissues, including 10 brain regions. Genetic variants that are associated with regulation of *GBA* were identified by screening 128 common SNPs located within the *GBA* locus for spatial eQTLs using CoDeS3D^30^ (Fig. 1A, Supplementary Fig. 1). The CoDeS3D^30^ algorithm tests for spatial eQTLs (GTEx database) that are supported by chromatin interactions (Hi‐C data), linking the common SNPs in the *GBA* locus with genes in *cis* (< 1 Mb between the SNP and gene). Of the 128 common SNPs, 56 (44%) tested were identified as *cis*‐acting spatial eQTLs that were associated with decreased *GBA* expression in 14 of 29 peripheral tissue types (Fig. 1B, green; Supplementary Table 2). Blood was the single exception, where the *cis*‐acting spatial eQTLs were associated with an increase in *GBA* expression (Fig. 1B). The *GBA* pseudogene *GBAP1* was also subject to *cis*‐acting spatial eQTLs and typically displayed the same response as *GBA* except in the blood and in the brain where *GBAP1* remained subject to *cis*‐acting regulation.

### Genetic Variants at the *GBA* Locus Regulate the Expression of Potential Modifier Genes

**FIG. 2.**
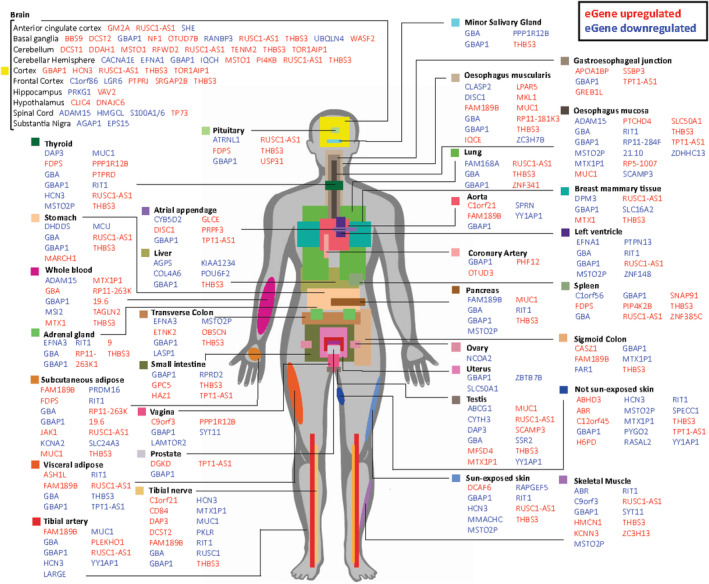
Single nucleotide polymorphisms within *GBA* are associated with tissue‐specific patterns of modifier gene expression. Single nucleotide polymorphisms within *GBA* are physically and functionally associated with the expression levels of 143 modifier genes across the 47 tissues. For example, single nucleotide polymorphisms in *GBA* form *trans‐*expression quantitative trail locus connections to *AGAP1* (100 Mb away from *GBA* on chromosome 1) and *EPS15* (chromosome 2) in the substantia nigra, both of which have strong neurodevelopmental‐relevant functions. Red, upregulated (normalized effect size is positive); blue, downregulated (normalized effect size is negative). [Color figure can be viewed at wileyonlinelibrary.com]

**FIG. 3.**
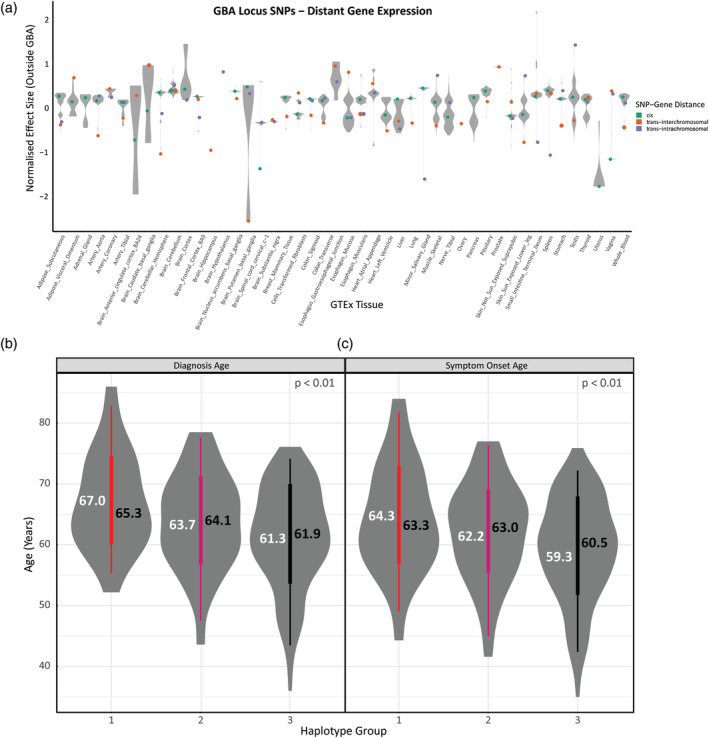
SNPs impact modifier gene expression and Parkinson's disease symptom onset and diagnosis age. (**A**) The distribution of normalised effect size for expression quantitative trail locus from SNPs within the *GBA* locus across the 47 the Genotype‐Tissue Expression (GTEx) project tissue types. (**B**) Parkinson's disease symptom onset age and (**C**) diagnosis age were significantly correlated to haplotype groups (*r* = 0.21 for onset and r = 0.23 for diagnosis, *P* < 0.01). Symptom onset and diagnosis age are highly collinear variables, and each show a decrease in mean (white) and median (black) age across the haplotype groups. SNPs, single nucleotide polymorphisms. [Color figure can be viewed at wileyonlinelibrary.com]

The 128 common SNPs at the *GBA* locus were then examined for their ability to regulate other genes, some of which might modify the *GBA* phenotype. In addition to *cis*‐acting spatial eQTLs, the CoDeS3D^30^ algorithm also detects *trans*‐acting spatial eQTLs (>1 Mb between the SNP and gene on the same chromosome, or the SNP and gene are on different chromosomes). In total, 73 SNPs regulate 143 potential modifier genes across many tissues (Fig. 2, Supplementary Fig. 2a). The ratio of *cis‐*connections to *trans‐*connections (4654: 288) we detected agrees with observations that long‐distance (*trans*‐acting) connections are less prevalent but more tissue‐specific than short‐range (*cis*‐acting) connections.^43^, ^44^, ^45^ The effect sizes of these spatial eQTLs vary largely by tissue as well as distance of connection (Fig. 3A). We additionally analyzed the 73 SNPs for transcription factor binding sites and enhancer/promoter markers using Haploreg version 4.1 ((https://pubs.broadinstitute.org/mammals/haploreg/haploreg.php). Notably, 38% (28 of 73) and 71% (52 of 73) of these SNPs display histone marks associated with promoter and enhancer activity, respectively (Supplementary Table 3).

### Modifier Genes Are Enriched for PD Relevance That Include Synaptic Vesicle Formation and Recycling

We hypothesized that if the *cis*‐acting and *trans*‐acting spatial eQTLs (centered on *GBA*) control modifier genes, then these genes should be epistatic with their products acting in the same or complementary pathways to the *GBA*‐encoded GCase. Therefore, we performed pathway analyses and literature data mining on the 143 potential modifier genes. Collectively, these analyses revealed that 24% (n = 35) of the potential modifier genes have an association with PD or a related pathway (Supplementary Fig. 2b, Supplementary Table 4). Of these genes, 4 (*DNAJC6* [*PARK19*], *SYT11, SNAP91* [*AP180*], *EPS15*) showed enrichment involving synaptic vesicle formation and recycling, with *DNAJC6* and *SYT11* previously associated with PD.^46^, ^47^, ^48^, ^49^, ^50^ Nine genes had known neuronal functions (Supplementary Table 4), and notably 23% (33 of 143) of the modifier genes were identified as being druggable targets (Supplementary Table 5).

Changes in the expression of mutationally constrained genes are likely to have a higher impact on fundamental disease processes than those that are not highly constrained.^51^ As such, the activity of genes that are intolerant to loss‐of‐function (LoF) mutations are more likely to be altered by changes to gene expression. In PD, it has been reported that there is a significant enrichment of heritability in LoF‐intolerant genes across almost all cell‐type classes.^52^ Therefore, we compared our *cis*‐acting and *trans*‐acting eQTL gene lists for enrichment for LoF intolerance (Supplementary Fig. 3a,b; intolerance defined as probability of being loss‐of‐function intolerant (pLI) ≥ 0.9).^53^ Comparing the gene set that is not regulated by variants within the GBA locus with the gene sets that are affected by *cis*‐eQTLs or *trans*‐eQTLs from variants inside *GBA*, a pattern of intolerance emerges. eQTLs at longer distances, on average, connect to a higher proportion of LoF‐intolerant genes. Those eQTLs connecting to genes outside of *GBA* containing chromosome 1 were even more biased toward intolerance, with a much larger proportion of genes with pLI > 0.9. To test for a threshold effect, we also looked for median pLI for each gene list, which is also much larger (0.83) for the longest (*trans‐*interchromosomal) connections than for the other gene lists (median pLI < 0.1 for all other lists). These results are consistent with the *trans‐*eQTLs we identified as being important for PD.

### *GBA* Haplotypes Stratify Idiopathic Patients by Age of Symptom Onset and Diagnosis

To assess the clinical impact of the *cis*‐acting eQTLs, we hypothesized that the pleiotropic effects of *GBA* locus SNPs may be explained by the combined action of these eQTLs as a haplotype. To test this, targeted Nanopore sequencing (all exons and introns of *GBA* on 8.9 kb amplicons) on a cohort of 229 patients with PD^41^ was performed, resulting in the identification of 55 individual haplotypes (Supplementary Tables 6 and 7). We then clustered the haplotypes into 2 haplotype clusters (Supplementary Fig. 4; haplotype cluster A = minor/alternative alleles, haplotype cluster B = majority/reference alleles). To distinguish the effect of missense mutation carriers from the idiopathic cases, we removed from further analysis individuals containing *GBA* coding mutations known to alter *GBA*/GCase function (rs2230288 [E365K], rs75548401 [T408M], rs147138516 [D179H], rs76763715 [N409S], rs146774384 [R78C], novel subvariant [L335]), leaving 208 patients with IPD (Supplementary Fig. 4, Supplementary Table 7).

Individuals were then organized into haplotype groups according to whether they were homozygous or heterozygous for haplotype cluster alleles. A total of 26 individuals were classified as haplotype group 1 as they were homozygous for alleles from haplotype cluster A (minor/alternative genotype alleles; Supplementary Fig. 4, Supplementary Table 7); 67 individuals were heterozygous (ie, 1 allele from each of haplotype clusters A and B) and classified as haplotype group 2. A total of 115 individuals were homozygous for haplotype cluster B (majority/reference alleles) and were classified in haplotype group 3 (Supplementary Fig. 4, Supplementary Table 7). We regressed haplotype groups against phenotypic measures of PD to determine if there was an association with clinical outcomes (Supplementary Table 8). We identified that symptom onset age and diagnosis age (highly collinear variables) were significantly correlated to haplotype groups (*r* = 0.18, *P* < 0.01; Fig. 3B, Supplementary Fig. 5) and that haplotype group 1 (predominantly SNP minor/alternative genotype alleles) had a mean diagnosis age 5.7 later than group 3 (Fig. 3B). The 3 SNPs (rs9628662, rs762488, rs2009578) within the haplotype that we identified as spatial eQTLs modulate peripheral *GBA* expression and the expression of potential modifier genes in either the brain and/or periphery (Supplementary Table 2).

### Distant Loci Regulate *GBA* Expression in the Substantia Nigra and Cortex

*GBA* expression in the substantia nigra was not associated with *cis‐*acting eQTLs (Fig. 2). However, *trans‐*acting eQTLs (SNPs associated with the expression of genes >1 Mb away or on other chromosomes) better identify regulatory networks, associated transcription factors, and tissue‐specific gene regulation.^43^, ^44^, ^45^ Therefore, we undertook a genome‐wide search (using 31,471,836 SNPs) to identify *trans‐*acting eQTLs that regulate *GBA* expression in PD‐relevant brain tissues (substantia nigra and cortex) and the cerebellum, which is not strongly associated with PD (Supplementary Table 9, Supplementary Fig. 1). We used the genome‐wide association study–derived threshold for common variants (minor allele frequency (MAF) > 5%) with exome‐wide significance (*P* < 1 × 10 − ^6^) for the identification of *trans‐*acting eQTLs because we were looking solely for regulatory impacts on *GBA* expression.^54^ We identified significant (*P* < 1 × 10 − ^6^) distal SNP–gene associations for 4 loci in the cortex, 2 loci in the substantia nigra, and 0 loci in cerebellum (Supplementary Table 9). Notably, the 6 loci containing these *trans*‐acting eQTLs are on different chromosomes from each other and *GBA*, eliminating any case for their common action on *GBA* expression being through correlation of effects in linkage disequilibrium (Table 2). Further to this, the vast majority of the eQTLs at these loci display transcription factor binding sites as well as histone marks associated with enhancer activity (Supplementary Table 8). Finally, CoDeS3D^30^ analysis identified the regulatory effect associated with the variants on chromosome 22 on *GBA* expression to be specific to the substantia nigra (Supplementary Table 10).

**TABLE 2.** Regulatory effect of variants located throughout the genome on *GBA* mRNA in the SN and cortex

Distal regulatory variants	Chr.	Effect on *GBA* mRNA in SN	Effect on *GBA* mRNA in Cortex	Regulatory Variant Gene	Location
rs4328731 + 13 other variants	22	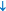		*ELFN2*	Intronic
rs2446914	8	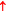		*RGS22*	Intronic
rs748178, rs736333, rs92481	3		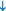	8kb 5' of RP11‐62G11.2	Intergenic
rs4781974	16		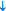	17kb 5' of XYLT1	Intergenic
rs117360313	6		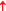	*GPRC6A*	Intronic
rs77317045	10		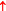	*ZNF365*	Intronic

Expression is: 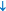, downregulated; 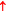, upregulated. *GBA* is located on Chr 1.

Abbreviations: Chr., chromosome; SN, substantia nigra.

## Discussion

We provided evidence that expands the role of the *GBA* locus to include its action as a hub of regulatory activity of importance to PD onset. We have demonstrated that the impact on *GBA* is not limited to missense mutations (eg, N370S, E326K, V394L, L444P) that directly impact the protein structure of the GCase but additionally include common noncoding variants that regulate its expression. *GBA* expression is significantly modulated by both *cis‐*acting eQTLs from within the *GBA* locus as well as by *trans*‐acting regulation from loci on different chromosomes. The regulation by *cis*‐acting spatial eQTLs modulates *GBA* expression in 14 peripheral tissues (including the heart, esophagus, stomach, pancreas, and salivary and adrenal glands), but not in the brain. Although peripheral, these tissues are highly regulated and innervated by the autonomic and enteric nervous systems, which are tightly associated with the brain. Reductions in *GBA* expression/GCase levels are inversely related to the accumulation of αsyn in Lewy body inclusions,^55^ and intriguingly, 5 of these peripheral tissues with spatial eQTLs decreasing *GBA* expression have to date been found to contain αsyn pathology in patients with PD (Supplementary Table 11).

We contend that reduced *GBA* expression in specific peripheral tissues renders them susceptible to αsyn accumulation and deposition in these tissues and may contribute to some of the nonmotor symptoms experienced by patients (eg, orthostatic hypotension, gastrointestinal dysregulation), thereby supporting the view of PD as a multisystem disorder. Furthermore, we speculate that this αsyn pathology in peripheral tissues (eg, stomach) may additionally contribute to the proposed gut‐to‐brain transmission of pathogenic αsyn observed in animal models.^56^, ^57^ Finally, an examination of these peripheral tissues may identify accessible peripheral tissues relevant to disease pathogenesis, potentiating early disease detection and therapy development.

The *cis*‐acting spatial eQTLs were also found to regulate 143 genes, including genes with published connections to PD. Some of these genes might modulate the disease process and progression as the 3 noncoding *cis* SNPs (rs9628662, rs762488, rs2009578) within the *GBA* locus form a haplotype that is significantly correlated with age of symptom onset and the age at diagnosis. Individuals who are homozygous for minor/alternate genotype alleles display a ~ 5‐year delay of symptom onset and diagnosis, while heterozygous individuals show an intermediate degree of disease delay (~2.9 years for onset and 2.4 years for diagnosis), suggestive of a transcriptional dose‐effect (Fig. 3B). Surprisingly, despite acting as spatial eQTLs, these SNPs do not regulate *GBA* expression in the brain. However, they do modulate the expression of other genes within the brain that include *DNAJC6* [*PARK19*], *MSTO1*, *NF1*, *PRKG1*, *RANBP3*, *RUSC1*‐*AS1*, *S100A1*, *THBS3* (Supplementary Table 2), some of which are PD associated (Supplementary Table 4). Moreover, these spatial eQTLs also regulate expression of *GBA* and other genes in peripheral tissues (Fig. 2). Therefore, the mechanism of the haplotype‐specific delayed disease onset/diagnosis could result from (1) altered expression of potential modifier genes in the central nervous system and/or peripheral tissues, (2) increased *GBA* expression in the blood, (3) decreased *GBA* expression in peripheral tissues other than blood, or (4) a combination of these.

The *trans* regulation of *GBA* expression in the brain was discovered by a genome‐wide search that identified significant distal SNP–*GBA* associations for 2 loci in the substantia nigra and 4 loci in the cortex (3 SNPs increased *GBA* expression, 3 SNPs decreased *GBA* expression; Table 2), with the 6 loci located on different chromosomes. We propose that the independent assortment of these common variants could produce variant combinations whose resulting spectrum of *GBA* expression levels in the substantia nigra or cortex significantly contributes to the variable motor and cognitive phenotypes of PD. For example, in *GBA* carriers, the coinheritance of common variants that enhance the expression of *GBA* could mitigate the phenotypic consequences of *GBA* mutations that reduce lysosomal GCase activity. Such circumstances could contribute to the incomplete penetrance of *GBA* mutations in which the majority of people who carry *GBA* mutations do not develop PD by the age of 80.^15^ The reverse would also be true with *trans*‐acting regulatory variants decreasing *GBA* expression in the substantia nigra or cortex and potentially exacerbating motor or cognition/dementia symptoms, respectively, in carriers of *GBA* mutations. Therefore, we contend that *GBA* expression is a key variable that on its own begins to explain the incomplete or reduced penetrance of GBA mutations.

Idiopathic patients lacking *GBA* mutations display reduced *GBA* expression and GCase activity in the substantia nigra for unknown reasons.^18^, ^19^ The common *trans‐*acting variants regulating *GBA* expression in this tissue are strong candidates to also account for this occurrence in that such patients might possess variants decreasing *GBA* expression. This observation raises the possibility of genotyping patients with IPD for these *trans‐*acting regulatory variants. This would enable identification and stratification of patients lacking *GBA* mutations but whose *GBA* locus regulatory variant combination is predicted to confer a significant decrease in *GBA* expression in the substantia nigra or cortex, which in turn may have distinct clinical features, an aspect we are currently investigating in patient whole genome sequence (WGS)‐clinical datasets. The subset of idiopathic patients with reduced *GBA* expression may benefit from therapeutics that increase GCase activity, including ambroxol, which has entered clinical testing because of its capability to improve function of GCase.^58^, ^59^

Looking beyond the implications of *GBA* in PD*,* mutations at this loci are also a major risk for the synucleinopathy DLB (odds ratio, 8.28).^27^ The clinical distinction between DLB and PDD is the relative onset timing of motor and cognitive symptoms. We have identified *trans*‐acting regulatory variants that independently affect *GBA* expression in either the substantia nigra or cortex, which are brain regions associated with motor and cognitive functions, respectively. Therefore, it is tempting to speculate on the relative contributions of these variants in modulating the sequence of motor or cognitive decline in some individuals. Interestingly, the *cis GBA* regulatory variants may also contribute to DLB as αsyn pathology is also observed in the peripheral tissues of DLB patients (Supplementary Table 11).

Mutations in *GBA* are causal for Gaucher disease, a potentially devastating disease that can impact the brain and peripheral tissues. Gaucher disease displays incomplete penetrance and significant variability in genotype–phenotype correlations. The factors that influence the tissues affected (central nervous system vs. peripheral), disease severity, or progression within particular genotypes are unknown. We contend the *cis‐*acting and *trans‐*acting eQTLs that we identified as regulating *GBA* and modifier gene(s) expression are excellent candidates to explain the phenotypic variability that is observed in Gaucher disease.

Our analyses identified a number of *cis‐*acting variants regulating *GBA* expression in many bodily tissues, yet these spatial eQTLs are not active within the substantia nigra or cortex. This is an intriguing and unexpected finding given the conventional view that PD is a disorder of the brain. One may speculate that the high regulatory activity of *GBA* eQTLs on modifier genes may be dampening the capacity for simultaneous transcription, thereby reducing the activity of such *cis*‐acting eQTLs within the brain. Interestingly, other genes known to have a strong tissue‐specific disease presentation, such as *BRCA1* (72% of women who inherit a harmful *BRCA1* mutation develop breast cancer by the age of 80^60^) also lack significant *cis‐*acting eQTLs in GTEx within the relevant (ie, breast) tissue. Genome‐wide association study (GWAS) SNP rs1799949, the only GWAS SNP mapped to *BRCA1* and in GTEx, has significant eQTLs for *BRCA1* in the liver, tibial artery, and esophagus. Rs1799949 is also linked to a GWAS study of menopause, not breast cancer susceptibility. It should also be noted here that the haplotype cohort, the Hi‐C cohort, and GTEx libraries are separate and nonhomogenous sources (cell lines and 2–3 different sources of tissues), with large age and gender differences between the Hi‐C and GTEx data. Despite this, the *cis*‐acting and a limited number of *trans*‐acting spatial eQTLs were supported by Hi‐C connections derived from neuronal‐relevant tissues (Supplementary Table 2). These findings highlight that a lack of *cis‐*acting regulation of a disease gene within the known disease tissue may not be uncommon, emphasizing the importance of *trans‐*acting eQTLs, particularly when looking at complex traits and diseases.^44^

## Conclusion

This work provides a new perspective on the genetic etiology of PD, expanding the role of *GBA* from the gene encoding the GCase to that of a central regulator and modifier of PD onset, with *GBA* expression itself subject to distant regulation. Although extrapolating changes in *GBA* expression to the activity of the encoded GCase is challenging, it remains likely that the regulatory changes we have identified contribute to alterations in the activity of the GCase and other enzymes in the brain and peripheral tissues. It is notable that the distant genes that are connected to *GBA* are enriched for LoF intolerance and therefore can only be subject to regulatory changes at the gene or protein levels. This provides a plausible explanation for the variable phenotypes observed in *GBA* centric Gaucher disease, PDD, and DLB. Empirical studies are still required to functionally validate the regulatory regions, enabling quantification of their regulatory effect. Once validated, variants may be used for disease stratification, leading to personalized drug selection and the possible development or repurposing of novel drugs.

## Author Roles

(1) Research project: A. Conception, B. Organization, C. Execution; (2) Statistical Analysis: A. Design, B. Execution, C. Review and Critique; (3) Manuscript: A. Writing of the first draft, B. Review and Critique.

W.S.: 1C, 3A

S.F.: 1C, 3A

T.F.: 1C, 3B

O.E.E.G.: 1C, 3B

T.L.P.: 1C, 3B

S.Q.: 3B

A.J.D.: 3B

J.K.P.: 3B

T.J.A.: 1C, 3B

M.A.K.: 1C, 3B

A.C.: 1A, 3A, 3B

J.M.O.: 1A, 1B, 3A, 3B

## Financial Disclosures (for the preceding 12 months)

W.S. received salary support from the Royal Society of New Zealand Marsden Fund. S.F. received PhD stipend from the University of Auckland. T.F. received salary support the Health Research Council of New Zealand. T.P. received financial support from NZBRI and the Neurological Foundation of New Zealand. A.D. received salary support from the University of Auckland and research grants from New Zealand and the United States. J.P. received salary support from the University of Auckland and research grants from New Zealand. T.A. received salary support from the University of Otago and Anderson Neurology Ltd. M.K. received salary support from the University of Otago. A.C. received salary support from the Garvan Institute of Medical Research and The Australian Parkinson’s Mission. J.M.O’S. received salary support from the University of Auckland. The study received research grants from New Zealand (HRC, RSNZ Marsden fund), and philanthropic donations from University of Auckland Foundation.

## Supporting information


**Supplementary Fig. 1** Methods workflow
All 128 common SNPs located within *GBA* (chr1:155204239‐155214653) were analysed for putative spatial regulatory connections. Briefly, the coding regions that each SNP (blue circle) within the locus was spatially connected to were identified. The resulting spatial SNP‐gene pairs were used to query GTEx for tissue eQTL interactions. In addition, 31,471,836 genome‐wide SNPs (orange circle) from dbSNP Human Build 151 were tested for an eQTL with *GBA*. All significant SNP‐gene associations were then analysed for pathway enrichment, drug‐gene interactions, haplotype clustering and Parkinson's disease relevance in the literature.

**Supplementary Fig. 2** a) Significant SNP‐gene spatial associations between SNPs in the *GBA* locus and distant genes (FDR p < 0.05). 2747 of those connections are nearby (*cis‐*, <1Mb from the SNP, green), 149 are distant on the same chromosome (*trans‐*, >1Mb on chr 1, orange), 139 are connections with genes on a different chromosome (purple).
b) Connections between variants in the *GBA* locus and distant genes reveal a number of genes with relevance to PD, located across the genome. Many literature‐reinforced spatial eQTLs are near *GBA* (*cis‐*, green), distant on the same chromosome (*trans‐*, orange), or on a different chromosome (*trans‐*, purple). SNPs in the distant *ELFN2* locus (chromosome 22) regulate *GBA* expression (pink).

**Supplementary Fig. 3** Probability of loss of function intolerance increases with distance from the GBA locus.
a) The proportion of genes that are LoF intolerant increases with distance from the GBA locus (pLI > 0.9), as defined by the gnomAD Consortium using their gene‐level constraint metric
b) The median pLI increases across the *cis‐* (green), *trans*‐intrachromosomal (distant on the same chromosome, orange), or *trans*‐interchromosomal (on a different chromosome, purple). Median is shown on plots.

**Supplementary Fig. 4** Hierarchical cluster analysis reveals two major haplotype clusters.
Haplotypes were clustered using Euclidean distance for the dissimilarity measure and Ward's minimum variance method, implementing Ward's clustering criterion (dissimilarities are squared before cluster updating). Cluster A (red) has alternate genotypes at the three SNPs of interest (rs9628662, rs762488, and rs2009578). Cluster B (black) includes the most prevalent haplotypes (including haplotype 1), and has mostly reference genotypes at all positions, including at the three SNPs of interest. rs3115534 was represented by the alternate allele in all individuals within our cohort. Haplotypes that contain variants that inactivate the *GBA* locus are annotated with the rsID of the inactivating variant.

**Supplementary Fig. 5** Haplotype group correlation analysis reveals a significant relationship between genotype and PD age of onset or diagnosis.
Regression of haplotype groups against PD phenotypes identified a significant correlation between haplotype and two phenotypes: symptom onset age and diagnosis age (both highly correlated to each other; r = 0.18, p < 0.01). NA = missing data, “X” = a non‐significant correlation.

**Supplementary Table 1** Hi‐C datasets used in this study
Supplementary Table 2 Tissue‐specific results from CoDeS3D significant (p < 1x10‐6) results from the GBA gene locus
Supplementary Table 3 Haploreg v4.1 analysis of rsID, including proteins bound, motifs changed, promoter and enhancer histone marks
Supplementary Table 4 Literature review of the eGenes, expression in brain (GTEx V7, 03/03/2019), and their main features as related to PD
Supplementary Table 5 Pathway analysis (KEGG), druggability analysis (DGIdb) and protein classification (Protein Atlas) of eGenes
Supplementary Table 6 The 55 Haplotype genotypes
Supplementary Table 7 Haplotype group designations by patient ID
Supplementary Table 8 Haplotype Groups and Phenotype data
Supplementary Table 9 Genome‐wide significant (p < 1x10‐6) associations with GBA in substantia nigra and cortex, and associated histone marks and motifs
Supplementary Table 10 Genome‐wide significant loci which associate to GBA regulation in substantia nigra and cortex are themselves regulatory of many other genes in a tissue‐specific manner
Supplementary Table 11 Alpha‐synuclein aggregates/pathology as observed in five tissues
