# Supplementary figures and images for: Common Variants Coregulate Expression of GBA and Modifier Genes to Delay Parkinson's Disease Onset
Source: Mov Disord. 2020 Jun 18;35(8):1346–56. doi: 10.1002/mds.28144 (PMC7496525; doi:10.1002/mds.28144)

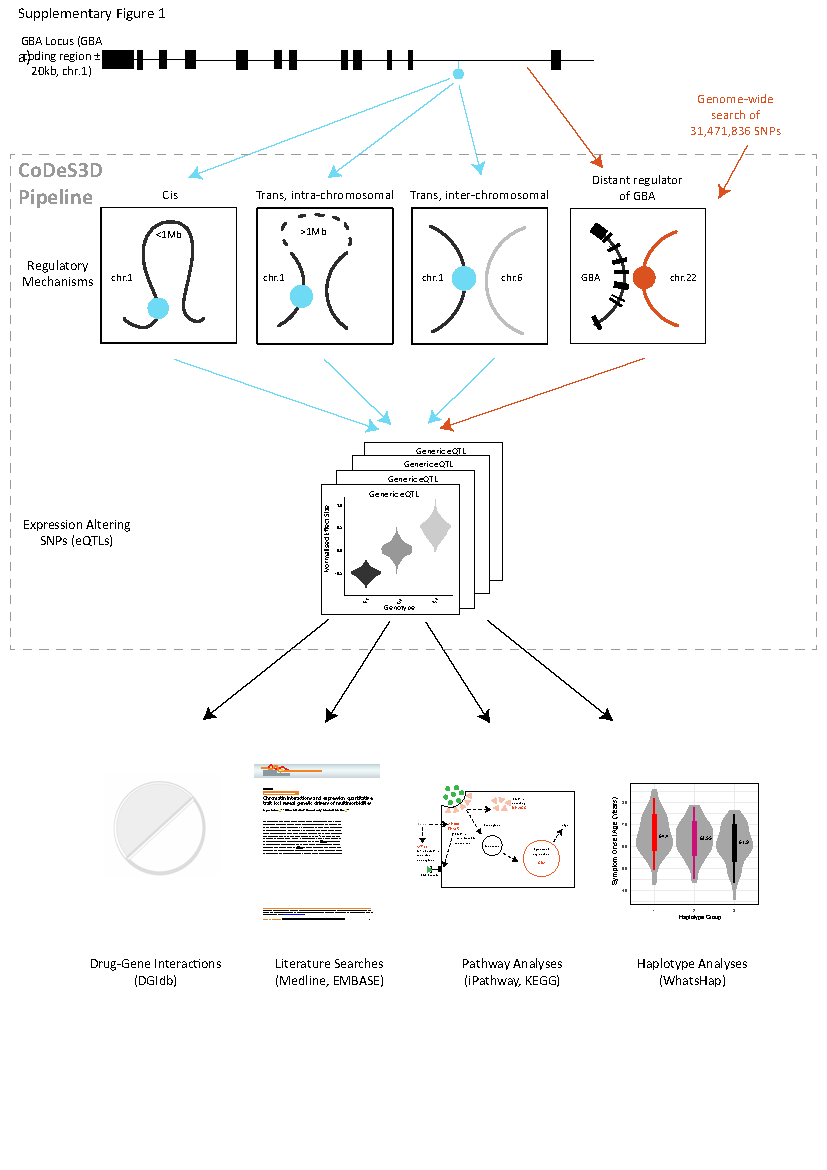

Supplement: Supplementary file 1 — Supplementary Fig. 1 Methods workflow All 128 common SNPs located within GBA (chr1:155204239‐155214653) were analysed for putative spatial regulatory connections. Briefly, the coding regions that each SNP (blue circle) within the locus was spatially connected to were identified. The resulting spatial SNP‐gene pairs were used to query GTEx for tissue eQTL interactions. In addition, 31,471,836 genome‐wide SNPs (orange circle) from dbSNP Human Build 151 were tested for an eQTL with GBA. All significant SNP‐gene associations were then analysed for pathway enrichment, drug‐gene interactions, haplotype clustering and Parkinson's disease relevance in the literature. [file MDS-35-1346-s001.tif]

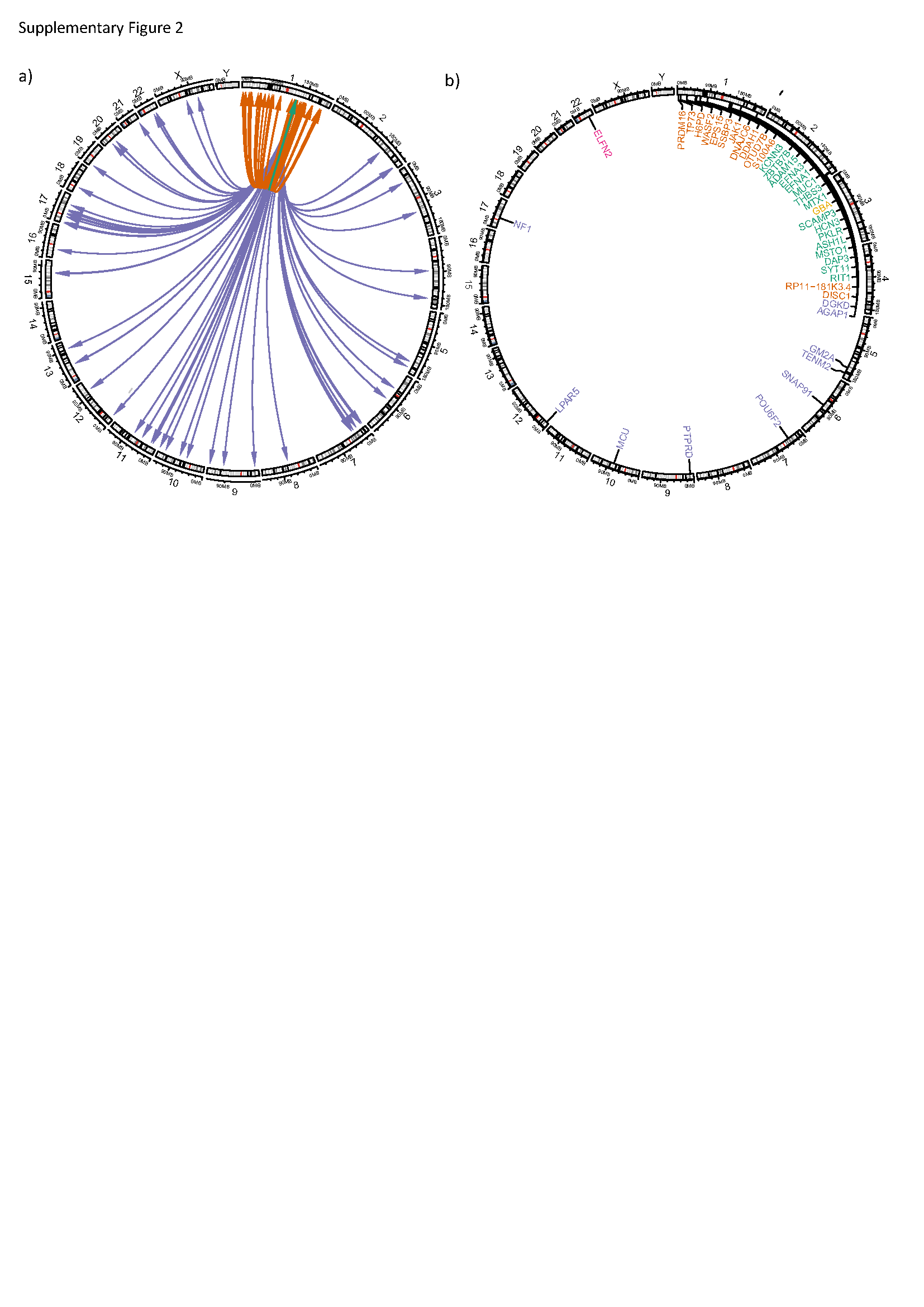

Supplement: Supplementary file 2 — Supplementary Fig. 2 a) Significant SNP‐gene spatial associations between SNPs in the GBA locus and distant genes (FDR p < 0.05). 2747 of those connections are nearby (cis‐, <1Mb from the SNP, green), 149 are distant on the same chromosome (trans‐, >1Mb on chr 1, orange), 139 are connections with genes on a different chromosome (purple). b) Connections between variants in the GBA locus and distant genes reveal a number of genes with relevance to PD, located across the genome. Many literature‐reinforced spatial eQTLs are near GBA (cis‐, green), distant on the same chromosome (trans‐, orange), or on a different chromosome (trans‐, purple). SNPs in the distant ELFN2 locus (chromosome 22) regulate GBA expression (pink). [file MDS-35-1346-s002.tif]

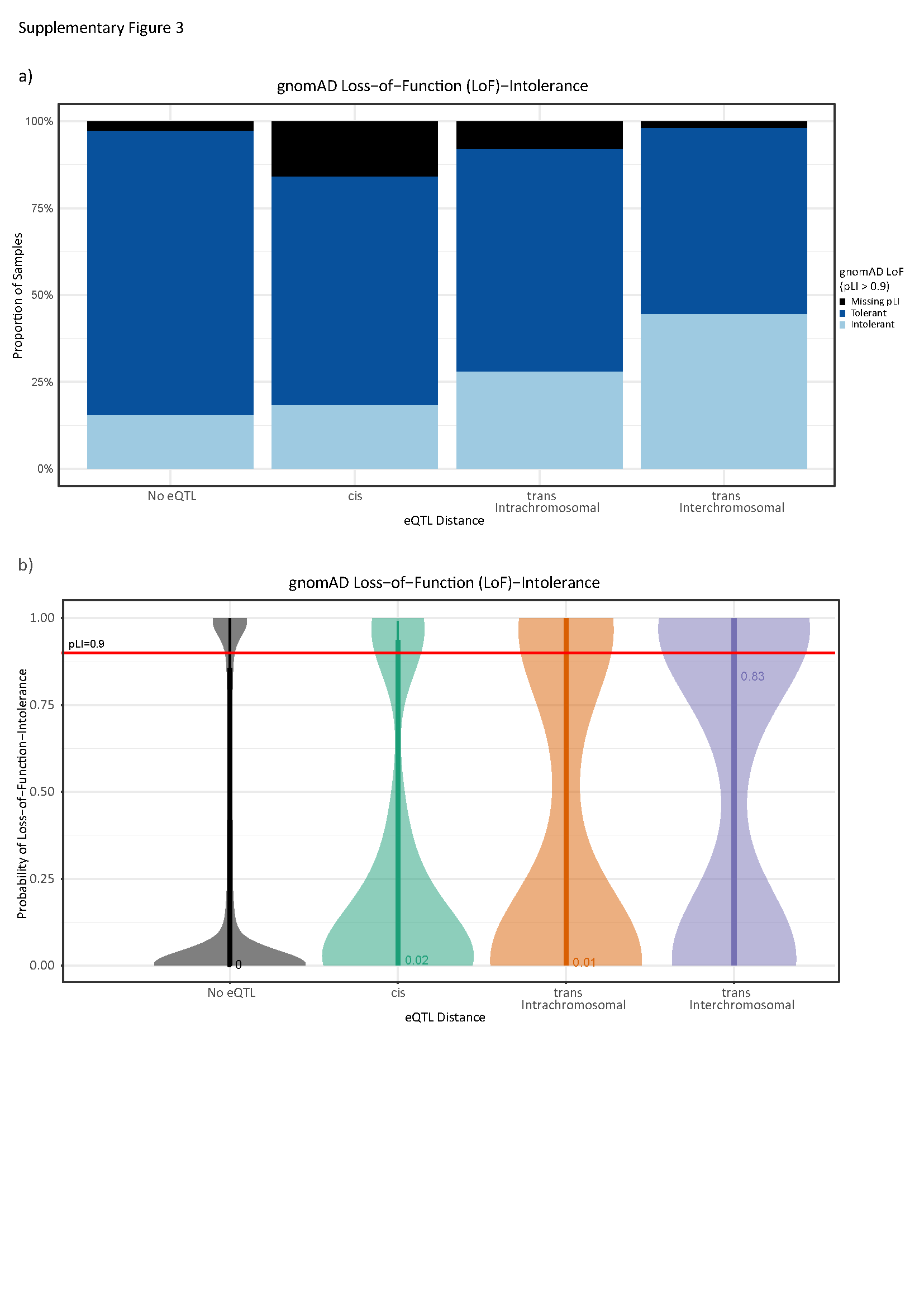

Supplement: Supplementary file 3 — Supplementary Fig. 3 Probability of loss of function intolerance increases with distance from the GBA locus. a) The proportion of genes that are LoF intolerant increases with distance from the GBA locus (pLI > 0.9), as defined by the gnomAD Consortium using their gene‐level constraint metric b) The median pLI increases across the cis‐ (green), trans‐intrachromosomal (distant on the same chromosome, orange), or trans‐interchromosomal (on a different chromosome, purple). Median is shown on plots. [file MDS-35-1346-s003.tif]

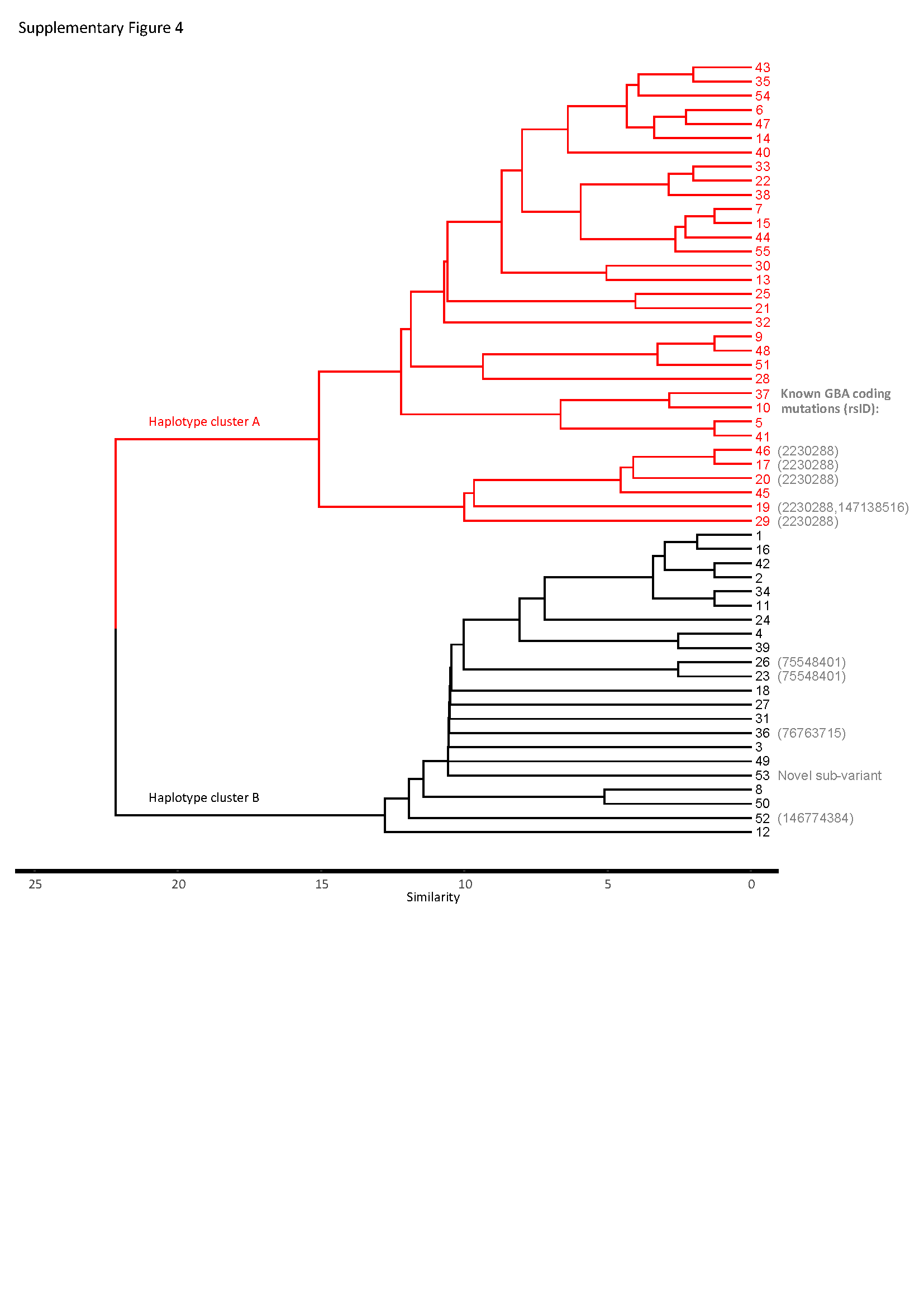

Supplement: Supplementary file 4 — Supplementary Fig. 4 Hierarchical cluster analysis reveals two major haplotype clusters. Haplotypes were clustered using Euclidean distance for the dissimilarity measure and Ward's minimum variance method, implementing Ward's clustering criterion (dissimilarities are squared before cluster updating). Cluster A (red) has alternate genotypes at the three SNPs of interest (rs9628662, rs762488, and rs2009578). Cluster B (black) includes the most prevalent haplotypes (including haplotype 1), and has mostly reference genotypes at all positions, including at the three SNPs of interest. rs3115534 was represented by the alternate allele in all individuals within our cohort. Haplotypes that contain variants that inactivate the GBA locus are annotated with the rsID of the inactivating variant. [file MDS-35-1346-s004.tif]

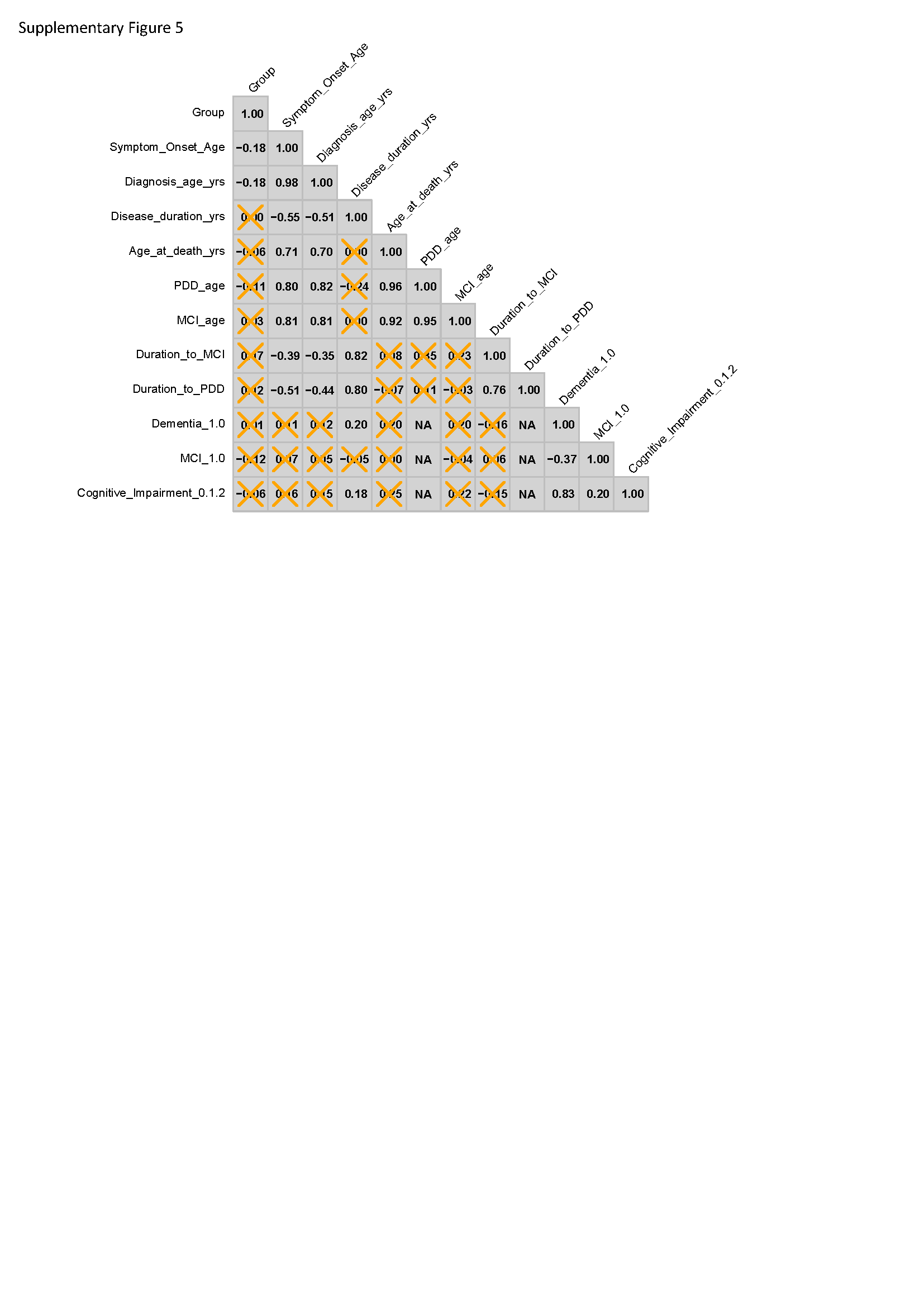

Supplement: Supplementary file 5 — Supplementary Fig. 5 Haplotype group correlation analysis reveals a significant relationship between genotype and PD age of onset or diagnosis. Regression of haplotype groups against PD phenotypes identified a significant correlation between haplotype and two phenotypes: symptom onset age and diagnosis age (both highly correlated to each other; r = 0.18, p < 0.01). NA = missing data, “X” = a non‐significant correlation. [file MDS-35-1346-s005.tif]
